# Disease of the Antrum of Highmore from a Medical Standpoint

**Published:** 1895-12

**Authors:** 


ARTICLE III.

### DISEASE OF THE ANTRUM OF HIGHMORE FROM A MEDICAL STANDPOINT.

### SYMPTOMS, SIGNS AND DIAGNOSIS.

In most cases I believe the trouble has been latent for a long time before they call in the physician or on the dentist for assistance. Persons may have the trouble for years before it announces itself. Jeanty, in reporting 22 cases of latent empyema, states that they were slow, insidious; there existed neither pain nor swelling in the affected cheek, and the sole manifestation of the disease was nasal secretion of continued or intermittent character, fetid or without odor of pus. The health of the patient becomes impaired if the disease has been of any great duration. They suffer from a mild degree of septicemia, from the absorption of pus. The classical symptoms are, as a rule, not present together —such as distension of the antrum, swelling of the cheek, infra-orbital pain, escape of pus, lying on the sound side.

It is only during the epidemic of influenza for the last two or three years that I can find any clear reports of acute attacks of antrum troubles. It is by studying these cases that we can prepare ourselves to make an early diagnosis of this class of troubles. I believe many cases occur that do not report to physicians, or, if they do, are not diagnosed. Felix Semon, the eminent laryngologist, reports† an acute case of his own antrum, caused by influenza. There was considerable coryza, a watery discharge from both nostrils, followed by a sensation of fullness in the left cheek, increasing to a sense of intolerable distension of the of the zygomatic region. The skin over the part became distinctly swollen and reddened and tender to the touch; temperature, 100.5. There was no frontal neuralgia. After these symptoms had lasted twenty-four hours, a violent blowing of the nose brought away an ounce of turbid greenish purulent fluid, followed by more of the same on lowering the head, inclined to the right. A few hours after this more of the same fluid escaped; later on a third escape took place. The acute complication now subsided, though two days later another discharge of greenish fluid of a mucoid character took place, which signalized the end of the affection. The points worthy of attention are: (1) The sudden and violent increase in pain during sneezing or coughing; (2) the limitation of pain to the affected region, and finally the tendency of influenza to single out the locality of its sequelle in different individuals. In Semon's case the sequelle observed occurred in the domain of the fifth nerve. Lennox-Brown and Moxham report acute cases with practically the same symptoms.

Wolfenstein, of Cleveland,* reports a case of acute empyema of the antrum of Highmore as follows: "The patient states that he caught a severe cold in his head two weeks ago, which ran an ordinary course till within the past three days, when he noticed soreness of the teeth,

---

†*British Medical Journal*, February, 24th, 1894.
**New York Medical Journal*, August 5th, 1893.

pain in the cheek of the left side; at the same time there was also a profuse discharge of pus from the left side of the nose; a feeling of soreness on this side of the nose also. The pain on the left side of the face is considerable, especially at night."

Kreig, in his account of acute causes, speaks of the great pain suddenly coming in the upper jaw, followed by swelling of the skin and a feeling of a weight in the antrum. Perforation gave great relief and a speedy cure. There were two relapses.

There is, in some cases, great pain in the upper jaw; the teeth seem to be long or pushed down out of their sockets, as it were. The pain shoots up in the ear, and at times takes on the nature of supraorbital neuralgia. The supraorbital pain is far more frequent than the infraorbital. The patients complain of a nasty odor, that is quite evident to themselves, but is not noticed by any one else; this odor is not constant, but in most cases can be observed by the patient drawing the mucus down and back in the posterior nares; this is caused by their drawing the odor out of the antrum during this action. In some cases the odor can only be noticed in the morning, and by others in the evening. This depends, I think, on the size and position of the opening in the antrum. Pus can be found, in most of the cases, coming out from behind the front of the middle turbinated bone. The flow of pus can be increased, in many cases, by holding the diseased side uppermost and the head forward and downward. The pus at the anterior end of the middle turbinated bone pulsates at times.

Zeim, in 1888, showed that blennerhea of the nose rarely occurs without disease of accessory cavities, and pointed out and proved that where this symptom occurs it is usually due to empyema of the antrum. We have headache, complained of mostly in the frontal region, though in one case it was occipital and passed away after the operation. The opening in the antrum from the nose will, in most cases, be covered by polyps, or there will be either polypoid degeneration of the mucus membrane of the middle turbinated bone or hypertrophy of the same. Denuded bone about the opening can very often be felt with a probe.

McBride calls attention to marked redness of the gum on the diseased side; this sign I have not met with. Pain on percussion will be found, in many cases, on the affected side, and when the teeth are struck. There is likely to be a feeling of increased fullness and distension, with an increase of pain on the affected side, headache and sickness of the stomach toward evening. This is caused by the position of the patient during the day being erect, which does not allow a discharge of pus to take place, the the antrum filling up full. Most of these cases are those, I think, where there is rather free drainage in the nose, and the symptoms or the trouble seems to be periodical. A case of this kind, now on hand, came to me for eye trouble; complained of neuralgia;, pain worse in the evening; fullness, not pronounced, on right side of face; severe headache; pain over the eye; pain so great at times as to cause sick headache and vomiting. After close questioning, patient admitted a nasty odor, noticed by herself only; pus free in the morning, less so at night, when the pain comes on; an abscess of a cuspid some four years ago; is wearing a rubber plate. Has a polypoid degeneration of the middle turbinated bone in the nose; pus flows from behind the bone, which is pushed firmly over to the septum.

Berger calls attention to the frequent occurrence of polyps and hypertrophy, which closes the opening of the sinus; the secreted liquid is retained in the cavity, where it serves for development of all kinds of bacteria, and particularly those which produce the mixture of trimethylamine, and dimethylamine, which one meets with in old red herring sauce, and the odor of which is identical with empyema.

One of the means for diagnosis is the electric lamp in mouth. In the healthy subject the pupil has a red reflex, and there is a bright, almond-shaped space under the eye. If these two are dark, there is a decided probability that there is trouble in the antrum. If with the dark pupil and dark space under the eye we have pain above the eye, or the whole side of the head is painful, and pus in the nose, we need not hesitate to make a diagnosis of pus in the antrum. The fact must not be overlooked that very often antrum troubles do not happen alone; they are, in most cases, associated with suppuration in the ethmoidal and sphenoidal cells and frontal sinus. This condition must be thoroughly treated at the same time as that of the antrum. Some have claimed that there is a resistance to the finger on percussion—almost dullness over the affected cheek; I have tried for this, but have not convinced myself of its correctness. Then, if we have a case with pus coming from one side of the nose, an odor that is only noticeable to the person himself, and this not at all times; the electric illumination, showing a dark space under the eye. With these three—or even with any two of them—we can safely make the diagnosis of pus in the antrum. If we are in doubt, the use of hydrogen dioxide (a few drops thrown up behind the middle turbinated bone and in the antrum) will, in most cases, decide the question. If pus is in the antrum, there will be felt a commotion, and the foaming caused by the decomposition of pus will be plainly seen under the middle turbinated bone. A small trocar and cannula can, for diagnosis, be thrust in the antrum, either through the canine fosse or through the external wall of the nose, under the inferior turbinated bone. The treatment of empyema of the antrum would seem to be simple and easy. We have a cavity filled with pus; all that needs to be done is to give it good drainage, keep it clean, and it will get well itself. This is simple in theory, but in practice does not seem to work. If we could see the cases soon after their commencement, the theoretical treatment would be successful; but we do not see the cases in this stage. It is only long after the beginning that we see them; then we have all the complications to combat. That antrum affections are troublesome to treat and bring to a recovery, we need only to refer to the great number of expedients and operations that have been performed and suggested for its relief in the last three or four years. The subject has been written on so much that one can say but little of anything that has not been said before. The treatment is, of course surgical: open the antrum; give good drainage. The best place for most cases to be opened is as is laid down by both Brown and Talbot, of Chicago, and Fletcher of Cincinnati, and is the point I have opened in the last cases I have had before I saw what these men had done; this is between the second bicuspid and first molar, and under the molar process. This place, Talbot and Fletcher have demonstrated, is the point where, in most every case, the lowest point of the antrum can be reached. After the antrum is opened it must be washed out and kept clean. Most any solution that is cleansing can and has been used; the changes have been wrung from warm salt water to a strong nitrate of silver solution. In one of the cases the man had used his own urine. To treat cases successfully all the complications must be removed, such as polyps in the nose; enlarged middle turbinated bones; pus in the ethmoidal cells and in the frontal sinus. These causes just mentioned, nose men are beginning to think, are the cause of most of the empyema of the antrum. This subject was discussed in England in December, 1894, and the majority were of this opinion. If there is a diseased tooth or necrosed alveolar process, these are the cases that dentists encounter; they must be removed. I hope when you, my dental friends here, do this, your cases may all do as well as one of your confreres says his cases do; that is, after removing the tooth and diseased bone, washing out thoroughly, they get well in two or three treatments.

The dentists, as a rule, and a great number of rhinologists, open in the cavity through the socket of an extracted tooth; this is a good way in a number of cases, especially if there is no complication. If the case has been of any duration there is likely to be polyps in the antrum or polypoid degeneration of the mucous membrane. If this happens, the condition must be removed by vigorous curetting or scraping of the cavity. We may have the mucous membrane of the cavity in folds. There may be bony septums growing in the antrum, the result of the inflammation, as was stated in the pathology. These must be broken down. The opening as described can be enlarged or an opening can be made through the cunine fosse. One thing that favors recovery is the fact that in pathological cases the opening in the nose is much larger than in normal cases. This trouble has been treated and cured by the injection of fluids through the opening in the antrum, through the inferior turbinated bone. A small trocar and canula, slightly bent, may be used, putting the point of the instrument well in the nose and beyond where the lachrymal duct enters the nose, and pushed in, out and upward, till it is felt to have entered a cavity. The trocar is withdrawn, and the cavity washed out. Kuster, of Berlin, advocates about the same way of operating. The nose is first put under the influence of cocain. Some operators open both through the inferior meatus and between the bicuspid and molar teeth; by this means they can easily wash out the antrum. The method by which the operation is done will depend on who discovers it—physician or dentist. I believe, though, there will in the future be fewer openings through the alveolar process, because we are discovering the trouble every day nearer its beginning. A number of men treat the cavity by the dry method. The cavity is cleaned by irrigating it well, drying it thoroughly by blowing in air, then iodoform, iodol or any antiseptic powder. Some brilliant successes have been had from this treatment. Packing the cavity after curetting with either bichloride or iodoform gauze is often done. But, whatever plan of treatment is decided on it must be thorough. The cavity must be made clean and kept clean. We have a good helper in cleansing in hydrogen dioxid or pyrozone. I would like to call attention to one little point in washing out the cavity—that is, do not use much force. Use a fountain syringe with plenty of fluid and do not have it raised very high above the head, and when the water is running in the antrum put the head in different positions, so that the fluid may reach all parts of the cavity. As to the instrument to open the cavity, there is nothing equal to the dental engine, but physicians and surgeons do not need to go to this expense. Any good bone drill will do the work; even a gimlet has been used. To keep the openings open various expedients have been used, the least irritating are either soft or hard rubber tubes made for this purpose. I have good results from gold tubes.

Although it is in the last few years that we have made the greatest advances in our knowledge of this condition, we are not very far in advance of the celebrated surgeon John Hunter in operating. He, over one hundred and twenty-five years ago, speaks of it in terms that would do to follow to-day. He says the first part of the cure, as well as that of all other abscesses, is to make an opening, not in the part where it threatens to point, for that would generally be through the skin of the cheek. If the disease is known early, before it has caused destruction of the fore-part of the bone, there are two ways of opening the abscess—one by perforating the partition between the antrum and the nose, which may be done, and the other by drawing the first or second grinder of that side and perforating the partition between the root of the alveolar process and the antrum, so that the matter may be discharged for the future by that way.—*Pacific Coast Dentist.*

